# The Biomarker Potential of the Neutrophil-to-Lymphocyte Ratio in Acute Exacerbation of Chronic Obstructive Pulmonary Disease

**DOI:** 10.7759/cureus.66750

**Published:** 2024-08-13

**Authors:** Bhumika Vaishnav, Ruchitha Pailla, Aniruddh Wadivkar, Harshitha Ummaleti, Nikhil I Doshi

**Affiliations:** 1 General Medicine, Dr. D. Y. Patil Medical College, Hospital and Research Centre, Dr. D. Y. Patil Vidyapeeth, Pune (Deemed to Be University), Pune, IND

**Keywords:** chronic obstructive pulmonary disease (copd), acute exacerbation of chronic obstructive pulmonary disease, cigarette smoking, lymphocytes, neutrophils

## Abstract

Background

Chronic obstructive pulmonary disease (COPD) is characterized by periods of exacerbations and seasonal variations due to the recruitment of inflammatory cells. Various cells, such as neutrophils, lymphocytes, and the inflammatory mediators released by them, play a role in its pathogenesis. The current study was conducted to determine the role of the neutrophil-to-lymphocyte ratio (NLR) as a marker in acute exacerbation of COPD (AECOPD).

Materials and methods

We conducted a cross-sectional observational study at a tertiary care center in Western Maharashtra over six months after receiving approval from the institutional ethics committee. The study included 50 patients with AECOPD and 30 age and gender-matched controls without COPD. The patients were examined with a detailed history, complete blood count, and spirometry. The NLR was calculated and patients’ disease severity was assessed using Global Initiative for Chronic Obstructive Lung Disease (GOLD) staging.

Results

The mean age in the AECOPD group was 67.5 ± 12.5 years, whereas it was 46.2 ± 18.5 years in the control group. There was a male predominance in the AECOPD group (32/50). The majority of patients were in the GOLD stage 2 (42/50), followed by GOLD stage 3 (7/50). A total of 28 out of 50 AECOPD patients required admission to the medical intensive care unit. In the AECOPD group, 39 were smokers, and 11 were nonsmokers. Smoking was significantly associated with AECOPD when compared with controls. The mean NLR was 10.9 ± 10.2 in AECOPD patients and 4.3 ± 3.1 in the controls. Among cases with high NLR (≥5), seven required mechanical ventilation, 13 required noninvasive ventilation (NIV), and six succumbed to death. The mean duration of mechanical ventilation in this group was 12.5 ± 4.5 days, and for NIV, it was 7.5 ± 5.5 days. Conversely, patients with lower NLR (≤5) were more likely to avoid artificial ventilation and had a better in-hospital outcome.

Conclusion

COPD exacerbations can harm a patient’s health, lead to disease progression, and increase mortality rates. Predicting exacerbations in advance can aid in early detection and treatment. NLR is an inexpensive, noninvasive, and easily available marker of inflammation and a predictor of outcome in AECOPD patients and hence should be calculated routinely in all patients with COPD.

## Introduction

Chronic obstructive pulmonary disease (COPD) is a condition characterized by marked limitation in airflow. Acute exacerbation of COPD (AECOPD) is a sudden worsening of patients’ baseline respiratory symptoms such as breathlessness, cough, and sputum with a history of exposure to risk factors such as smoking and other occupational exposure [1]. The cost of healthcare escalates during these frequent exacerbations often placing strain on intensive care unit (ICU) care. Exploring the root cause of exacerbations reveals heightened inflammation as a pivotal factor. This increased inflammation is due to various infections (bacterial, viral, or combined viral/bacterial) and environmental factors. Early detection and proper management of AECOPD are essential to avoid dangerous complications and mortality. Neutrophils, lymphocytes, and macrophages are the inflammatory cells responsible for the exacerbation. Repeated infections causing intermittent exacerbations lead to an ongoing chronic inflammatory state [2]. Neutrophils play an important role because they are an important source of proteases such as neutrophil elastase, and matrix metalloproteinases which cause injury to the lung parenchyma and airway [3]. Absolute counts of these cells, along with their ratio, reflect the acute or chronic inflammatory state.

The neutrophil-to-lymphocyte ratio (NLR) has been used as a marker of inflammation in numerous cancers, including breast, esophageal, pancreatic, and colorectal [4]. Because inflammation is the basis of the pathophysiology of lung parenchymal changes in COPD, the NLR can become a reliable and easily available prognostic tool for predicting immediate in-hospital survival in COPD. We aimed to study the NLR in AECOPD and delineate its role as a marker of immediate in-hospital outcomes.

## Materials and methods

Study design

This was a cross-sectional observational study conducted on 50 patients diagnosed with AECOPD. We carried out the study in the medical ICU of a tertiary care hospital in Maharashtra, India, from December 2023 to May 2024, following Institutional Ethics Committee approval (Ethics committee letter no.: IESC/PGS/2022/08).

Inclusion criteria

The study included patients above 18 years of age who were diagnosed with COPD (FEV1/FVC ratio < 0.7 on spirometry) or already had known cases of COPD.

Exclusion criteria

The study excluded patients under 18 years of age; those with chronic kidney or liver disease; those with a history of blood transfusion in the past three months; those with recent acute myocardial infarction, stroke, autoimmune disorders, and myeloproliferative disorders; and those undergoing chemotherapy. The diagnosis of AECOPD was based on risk factors and clinical symptoms, including a sustained increase in cough, sputum production, and dyspnea for more than 24 to 48 hours. This was followed by confirmation with spirometry (when the patient’s condition improved) and ensuring no other alternative reason for the clinical features and airflow limitation. We divided patients into two groups: 50 AECOPD patients and 30 age- and gender-matched healthy controls.

Data collection

The study collected demographics and clinical data such as age, gender, smoking history, and comorbidities. Laboratory data included a complete hemogram, with total leukocyte count, differential leukocyte count (neutrophils, basophils, lymphocytes, monocytes, and eosinophils), platelet count, and C-reactive protein (CRP). We calculated the NLR as the ratio of absolute neutrophil count (ANC) to absolute lymphocyte count. We categorized patients into two groups based on their NLR: NLR ≥ 5 and NLR ≤ 5.

Spirometry parameters, such as forced vital capacity (FVC), forced expiratory volume in 1 second (FEV1), and the ratio of FEV1 to FVC, were measured before discharge in patients who clinically improved and could perform the spirometry. Clinical outcomes like ICU admission, ward admission, types of mechanical ventilation, duration of artificial ventilatory support, and ICU outcomes (death or discharge) were noted.

Statistical analysis

We entered the collected data into an Excel sheet (Microsoft® Corp., Redmond, WA, USA) and analyzed it using Statistical Package for the Social Sciences (IBM SPSS Statistics for Windows, IBM Corp., Version 27.0, Armonk, NY) and expressed numerical values as mean ± standard deviation and expressed categorical values in numbers. We used the student's t-test for continuous variables and the Fisher's exact and Chi-square tests for categorical variables. The tests were two-tailed with a confidence interval of 95%. Results were considered statistically significant if p < 0.05.

## Results

The study included a total of 80 patients - 50 with AECOPD (henceforth known as cases) and 30 healthy controls. Among the 50 AECOPD cases, there were 32 (64%) men and 18 (36%) women. The mean age of the cases was 67.5 ± 12.5 years, and the mean age of the controls was 46.2 ± 18.5 years. The mean body mass index (BMI) of participants was significantly lower in controls compared to the cases (student's t-test p < 0.05). In the AECOPD group, 39 were smokers, and 11 were nonsmokers. The AECOPD group had a significantly higher number of smokers compared to the control group (Fisher's exact test, p < 0.05). Many cases (26/50) had a history of occupational exposure in the form of chemical fumes, dust, smoke, and exposure to biomass gases (Table 1).

**Table 1:** Demographic and lab parameters in study subjects Data presented as n (%) or mean ± standard deviation. BMI: body mass index; FEV1: forced expiratory volume in 1 second; FVC: forced vital capacity; GOLD: Global Initiative for Chronic Obstructive Lung Disease; COPD: chronic obstructive pulmonary disease; AECOPD: acute exacerbation of chronic obstructive pulmonary disease

Parameter	AECOPD cases, n=50	Controls, n=30
Age (years)	67.5 ± 12.5	46.2 ±18.5
Gender		
Men	32 (64%)	18 (60%)
Women	18 (36%)	12 (40%)
BMI (kg/m^2^)	29.1 ± 4.2	24.3 ± 3.9
Smoking as a risk factor		
Smokers	39 (78%)	0
Non-smokers	11 (22%)	30 (100%)
Other risk factors for COPD		
Biomass	10 (20%)	-
Passive smoking	8 (16%)	4 (13%)
Occupational exposure	26 (52%)	-
Past history/exposure to tuberculosis	4 (8%)	1 (33%)
Laboratory parameters		
Total leukocyte count (TLC) (cells/µL)	15808 ± 5067	8006 ± 2534
Absolute neutrophil count (ANC) (cells/µL)	12919 ± 4282	5500 ± 2368
Absolute lymphocyte count (ALC) (cells/µL)	1937 ± 2353	1594 ± 751
Neutrophil-to-lymphocyte ratio (NLR) on admission	10.9 ± 10.2	4.3 ± 3.1
NLR on discharge	6 ± 5.4	4.2 ± 2
C-reactive protein (CRP)	64.9 ± 30	4.29 ± 44
FEV1/FVC	57.9 ± 10	80.2 ± 2.5
GOLD staging		
Stage 1	0	-
Stage 2	42 (84%)	-
Stage 3	7 (14%)	-
Stage 4	1 (2%)	-

The mean ANC in AECOPD (cells/µL) was 12919 ± 4282 cells/µL. The mean NLR was 10.9 ± 10.2 in the AECOPD group compared to the control group which was 4.3 ± 3.1. Total leucocyte count, ANC, NLR, and CRP were significantly higher in the AECOPD group compared to the control group. There was a statistically significant correlation between Global Initiative for Chronic Obstructive Lung Disease (GOLD) stage 2 (42/50 patients) and NLR >5 in the AECOPD group (Table 1).

A high NLR ≥5 significantly correlated with the need for both mechanical and non-invasive ventilation (NIV) in ICU patients. Conversely, patients with a lower NLR (≤5) were more likely to avoid ventilation. However, the in-hospital outcomes (death vs discharge) were not significantly different between AECOPD cases with high vs low NLR (Table 2).

**Table 2:** In-hospital complications and outcomes according to the NLR in AECOPD patients A p-value < 0.05 is considered significant. ICU: intensive care unit; NLR: neutrophil-to-lymphocyte ratio; AECOPD: acute exacerbation of chronic obstructive pulmonary disease

Parameter	Frequency (%)	NLR ≥ 5	NLR ≤ 5	p-value
ICU admission	28 (56)	22 (79%)	6 (21%)	>0.05 (Chi-square)
Ward admission	22 (44)	14 (64%)	8 (36%)	
ICU admission				<0.05 (Fisher's exact test)
Invasive mechanical ventilation	7 (25)	7 (100%)	0	
Non-invasive mechanical ventilation	13 (46)	13 (100%)	0	
none	8 (29)	2 (25%)	6 (75%)	
Duration of artificial ventilatory support (days) - mean ± standard deviation				
Invasive mechanical ventilation	12.5 ± 4.5	-	-	
Non-invasive mechanical ventilation	7.5 ± 5.5	-	-	
ICU outcome				0.2 (Fisher's exact test)
Death	6 (21)	6 (100%)	0	
Discharge	22 (79)	16 (73%)	6 (27%)	

## Discussion

Long-term exposure to factors such as smoking, toxic substances, and inhalational agents in the environment results in a higher prevalence of COPD in the older adult group. The demographic analysis of our study also revealed that the mean age of patients in the AECOPD group was 67 ± 12 years, with a noticeable male predominance, consistent with findings by Gunay et al., who reported higher exacerbations in older men [5]. The mean BMI of participants was significantly lower in controls compared to the AECOPD group indicating that obesity is linked to the declining functional capacity of the lung [6]. Among the AECOPD patients, 78% were smokers, showing the critical impact of smoking on COPD exacerbations, as Levin et al. highlighted [7]. The highest number of patients in the AECOPD group were laborers which Silver et al. explained; they found that significant and ongoing exposure to environmental pollutants has a direct relation to the etiopathogenesis of COPD [8].

COPD has multifactorial etiopathogenesis with smoking and exposure to dust and noxious gases being the prime risk factors. Cigarette smoke contains high concentrations of oxidants, which induce inflammation in the airways, leading to chronic inflammatory changes and structural remodeling thereby obstructing airways [9].

COPD is a treatable and curable illness marked by persistent expiratory airflow restriction that is not completely reversible. It is a progressive condition characterized by an elevated and sustained inflammatory reaction to harmful gases and toxic chemicals in the airway mucosa. Although COPD begins with a complicated inflammatory response in the respiratory tract and lung tissue, it eventually develops into a systemic inflammation-related illness [10]. Several explanations have been offered to explain the rise in systemic inflammation caused by COPD. According to some researchers, inflammatory mediators flow across the pulmonary compartment [11]. Another theory has suggested that tissue hypoxia triggers an inflammatory response. The pro-inflammatory bacterial component lipo-polysaccharide also aggravates this response [12].

Elevated white blood cell (WBC) counts have been linked to an increased risk of repeated exacerbations. Our results showed that the total leukocyte count, neutrophil percentage, and ANC were significantly higher in the AECOPD group than in controls. This is consistent with the findings of Şahin et al. [13].

The NLR is a straightforward metric derived from a patient’s complete blood count, calculated by dividing the ANC by the absolute lymphocyte count. Consequently, any factors influencing these counts affect the ratio, which might increase or decrease. Lymphocytes, which play a role in regulating chronic inflammation and immune response, decrease during severe inflammation, further elevating NLR.

In the current study, we found that NLR was significantly higher in AECOPD than in controls, and in those AECOPD cases requiring ICU admission. Numerous studies have reached similar conclusions [14,15]. NLR is a well-established marker of inflammation that has been linked to various inflammatory disorders. COPD exacerbations are characterized by a flare-up of the chronic inflammatory process at both pulmonary and systemic levels. Exacerbations caused by bacterial, viral, and non-infectious agents promote increased airway inflammation and the release of pro-inflammatory cytokines and chemokines. These inflammatory markers are frequently elevated in AECOPD, resulting in increased systemic inflammation through neutrophil recruitment and activation, leading to a higher NLR in AECOPD compared to controls.

NLR can thus be a potential marker for disease severity and prognosis in COPD, corroborating the findings of Sakurai et al. [16]. The significant elevation in NLR during exacerbations aligns with the understanding that inflammation plays a pivotal role in COPD exacerbations.

The GOLD staging system is a critical tool for classifying COPD severity based on spirometry values, specifically the FEV1/FVC ratio. Our study included patients across various GOLD stages, predominantly in stages 2 and 3. As COPD progresses to higher GOLD stages, the inflammatory burden increases, which can further worsen the NLR. Research has shown that patients with higher GOLD stages have increased mortality and worse clinical outcomes​ [14]. NLR can be used to predict COPD exacerbations, and a higher NLR in regularly monitored patients suggests a higher likelihood of future exacerbations. A cheap and easily available marker such as NLR can indicate the severity of exacerbations without the need for spirometry [17].

CRP is another inflammatory marker that we measured in our study. We observed that CRP levels were significantly elevated in the AECOPD group compared to controls. Elevated CRP levels indicate systemic inflammation and are often associated with AECOPD. Studies have shown that higher CRP levels are linked to worse clinical outcomes, including increased hospital admissions and mortality. The combined measurement of CRP and NLR can provide a more comprehensive assessment of the inflammatory status and help in predicting exacerbations and outcomes in COPD patients.

The difference in the need for mechanical ventilation versus NIV among AECOPD patients can be attributed to the severity of inflammation and respiratory distress. Mechanical ventilation is often required for patients with more severe respiratory failure and systemic inflammation, indicated by higher NLR values. These patients are at a greater risk of complications and require more intensive respiratory support. In contrast, NIV is typically used for patients with less severe respiratory distress and lower systemic inflammation, as indicated by lower NLR values. NIV can effectively reduce the work of breathing and improve gas exchange without the need for invasive procedures, making it suitable for patients with moderate exacerbations.

Numerous studies have underlined the predictive relevance of neutrophils in the systemic circulation of COPD patients [18]. Celli and Barnes revealed that higher counts of WBC and neutrophils were solely and strongly associated with increased morbidity and mortality [19]. Furthermore, we observed that patients with higher NLR (≥5) had a higher requirement of mechanical and NIV in the ICU; however, it did not affect the in-hospital outcome.

The mechanism behind acute exacerbations explains the association between NLR and the severity of the disease. Increased neutrophils in the lung release various proteolytic enzymes from their granules, such as elastase and matrix metalloproteinases, which are implicated in emphysema. Consequently, airway neutrophilia has been found to have an inverse relationship with lung function. Our findings indicate that the NLR value reliably predicts future exacerbations. A larger sample size and follow-up studies for all AECOPD patients would further consolidate the role of NLR as a predictor for prognosis in COPD. In summary, NLR is a simple peripheral blood test, which can be used to assess the severity and activity of COPD.

Limitations

The study was conducted on a relatively small sample size of 50 AECOPD patients. A larger sample size might provide more robust and generalizable results. The study focuses on immediate in-hospital outcomes without considering long-term outcomes and complications that might arise post-discharge.

## Conclusions

COPD exacerbations can harm a patient’s health, lead to disease progression, and increase mortality rates. Predicting exacerbations in advance can aid in early detection and treatment. NLR is an inflammatory biomarker that increases dramatically during COPD exacerbations. Incorporating NLR measurement into routine clinical practice can help healthcare providers predict and manage these exacerbations more effectively.
